# Reversible Changes of Left Atrial Function during Pregnancy Assessed by Two-Dimensional Speckle Tracking Echocardiography

**DOI:** 10.1371/journal.pone.0125347

**Published:** 2015-05-01

**Authors:** Guang Song, Jing Liu, Weidong Ren, Wei Qiao, Jing Zhang, Ying Zhan, Wenjing Bi

**Affiliations:** 1 Department of Ultrasound, Shengjing Hospital of China Medical University, Shenyang, China; 2 Department of Obstetrics, First Affiliated Hospital of China Medical University, Shenyang, China; Temple University, UNITED STATES

## Abstract

**Background:**

Left ventricular diastolic function is impaired during pregnancy. However, changes in left atrial (LA) function remain unclear. We aimed to evaluate changes in LA function during pregnancy using two-dimensional speckle tracking echocardiography (2DSTE).

**Methods and Results:**

50 pregnant and 50 healthy nulliparous (control group) women were enrolled in this study. All pregnant women were followed up postpartum in sixth-month. The LA maximum volume, LA minimal volume and LA preatrial contraction volume were obtained using biplane modified Simpson’s method. LA filling volume, LA expansion index, LA ejection fraction, passive volume, passive emptying index, active volume, and active emptying index were calculated. LA longitudinal systolic strain (SS), systolic strain rate (s-SR), early diastolic strain rate (e-SR), and late diastolic strain rate (a-SR) were obtained by 2DSTE. Compared to the control group, the reservoir function was increased in pregnant patients (P<0.05); conduit function was decreased in pregnant patients (P<0.05); booster pump function was increased in pregnant patients (P<0.05). There was no statistically significant difference between the control group and postpartum group.

**Conclusions:**

LA reservoir and booster pump function were increased, while conduit function was decreased during pregnancy using 2DSTE. The changes were reversible. 2DSTE can easily assess LA function during pregnancy with good repeatability.

## Introduction

During pregnancy, the cardiovascular and other physiologic systems develop certain adaptive changes. The marked increase in volume load and metabolism in the third trimester worsens the potential for heart disease [1]. Many studies have demonstrated changes in left ventricular (LV) function during pregnancy, especially diastolic function [2, 3]. Although we know that left atrial (LA) structural and functional remodeling is a barometer of LV diastolic dysfunction, few studies have revealed the changes in the LA [4]. Some studies only focused on LA ejection fraction (LAEF) [5].

The LA serves multiple functions, acting as: 1) a reservoir during LV systole; 2) a conduit for blood transit from the pulmonary veins to the LV during early diastole; 3) a booster pump (active contractile chamber) that augments LV filling in late diastole [6]. Also, LA function is an important predictor of multiple adverse cardiovascular events, including death [7]. LA function can be obtained and expressed as LA volume, or global longitudinal strain and strain rate [8]. None of these three functions of the LA, or changes in LA strain and strain rate during normal pregnancy have been revealed in former studies.

Two-dimensional speckle tracking echocardiography (2DSTE) is a new technology to evaluate LA function using strain and strain rate in normal subjects [9]. Thus, we aimed to evaluate the changes in LA function during pregnancy using conventional echocardiography and 2DSTE.

## Materials and Methods

### Objective

Between February 2012 and October 2014, 50 gravid patients with an average gestational age of 35.8 (range 34–39) weeks were enrolled in this study based at Shengjing Hospital of China Medical University, and were followed up postpartum in sixth-month. The control group consisted of nulliparous women, matched for age and body size from the medical examination center. All subjects were healthy, yellow race Asians. Exclusion criteria were as follows: diabetes, hypertension, cardiovascular disease and multifetal gestation.

### Ethics

The China Medical University Ethics Committee approved this study. Written informed consent was obtained from all participants.

### Echocardiography Evaluation

Echocardiographic evaluation was performed in the left lateral position using a Vivid 7 (GE Healthcare, USA) and a 1.5/5 MHz phased array probe with a frame rate of 60–90 fps. All images and measurements were obtained from standard views according to the recommendations of the American Society of Echocardiography for chamber quantification [10]. All images were digitally stored and analyzed offline using customized software (EchoPAC, GE Healthcare).

### Parameters of LV function

LV end-diastolic dimension (LVDd) was obtained in the parasternal long-axis view. LV end-diastolic volume (EDV) and end-systolic volume (ESV) were obtained using the biplane modified Simpson’s method. Stroke volume (SV), cardiac output (CO), and LV ejection fraction (LVEF) were used as standard indexes of LV systolic function. The peak of early diastolic velocity (E wave) and peak of late diastolic velocity (A wave) across the mitral valve were obtained. The ratio between them (E/A) was used as a standard index of LV diastolic function.

The Tissue Doppler imaging indices peak velocity of early (E´) and late (A´) diastolic filling were measured at the level of the mitral septal annulus and lateral annulus on the apical four-chamber view.

### Conventional echocardiography parameters of LA function

The LA anteroposterior dimension (LAAD) was obtained in the parasternal long-axis view. In accordance with previous study [11], the following parameters were obtained using the biplane modified Simpson’s method: 1) LA maximum volume (LAVmax), obtained from an end-systolic frame just before mitral valve opening; 2) LA minimum volume (LAVmin), obtained from an end-diastolic frame just before mitral valve closure; and 3) LA preatrial contraction volume (LAVpre-a), obtained from the last frame just before mitral valve reopening as the result of LA contraction. Each of these parameters was corrected by body surface area (BSA).

LA reservoir function was assessed by: 1) LA filling volume, calculated as LAVmax—LAVmin; 2) LA expansion index, calculated as (LAVmax—LAVmin) / LAVmin×100; and 3) LA diastolic emptying index, calculated as (LAVmax—LAVmin) / LAVmax×100, just as same as LA ejection fraction (LAEF).

LA conduct function was assessed by: 1) passive atrial stroke volume, calculated as LAVmax—LAVpre-a; 2) passive emptying index, calculated as (LAVmax—LAVpre-a) / LAVmax×100; and 3) LA conduit volume, calculated as the difference between SV and LA filling volume.

LA booster pump function was assessed by: 1) active atrial stroke volume, calculated as LAVpre-a—LAVmin; and 2) active emptying index, calculated as (LAVpre-a—LAVmin) / LAVpre-a×100.

### Strain and strain rate of LA using 2DSTE

**Fig 1:**
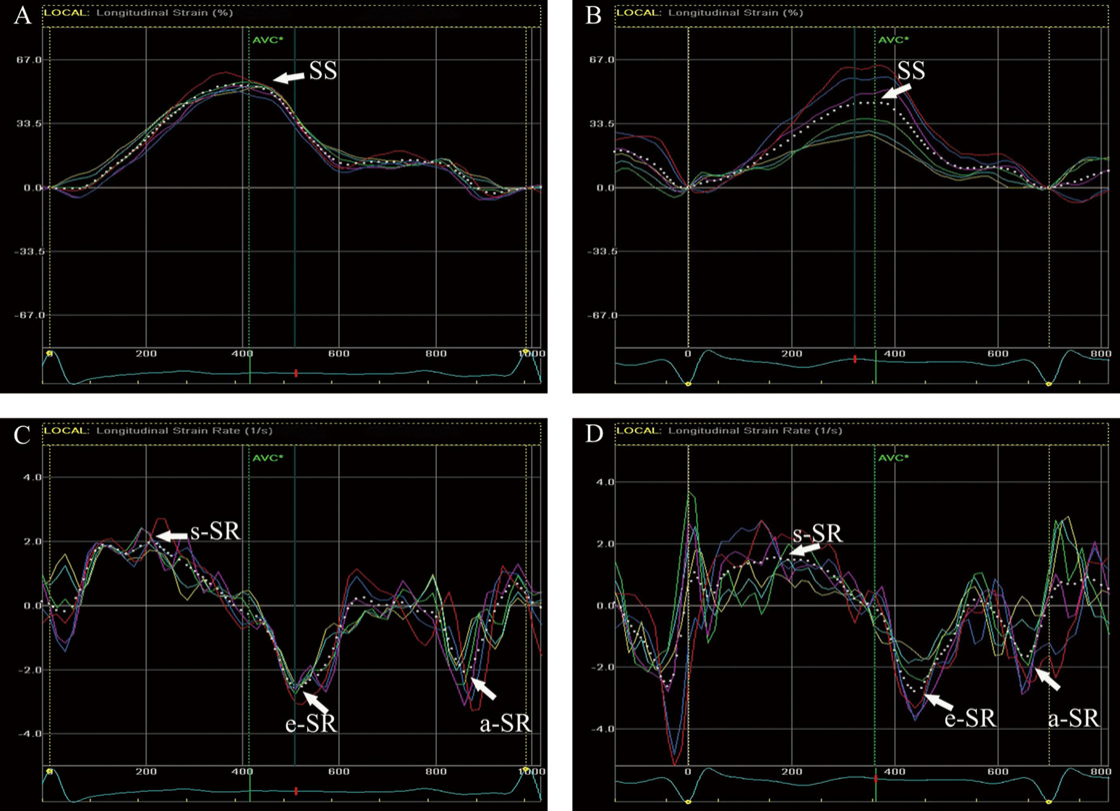
LA Strain and strain rate obtained by 2DSTE in the pregnant (A, C) and control group (B, D). LA = left atrial; SS = systolic strain; s-SR = systolic strain rate; e-SR = early diastolic strain rate; a-SR = late diastolic strain rate.

Strain and strain rate of LA were analyzed by two-dimensional speckle tracking software (EchoPAC, GE Healthcare). One cardiac cycle was analyzed between two contiguous R-waves of the ECG in the apical four-chamber view. The endocardial boundary of the LA was delineated manually; the software automatically drew the epicardial boundary. The width of the region of interest was manually adjusted when necessary. The software automatically divided the LA wall into 6 segments, then generated curves of global longitudinal strain and strain rate of LA. The peak of systolic strain (SS), systolic strain rate (s-SR), early diastolic strain rate (e-SR), and late diastolic strain rate (a-SR) were obtained from curves in different phases (Fig 1).

### Statistical analysis

All parameters are expressed as the mean ± standard deviation. The unpaired t-test was performed between the control group and two others. The paired t-test was performed between the pregnant and postpartum groups. Pearson correlation analysis was performed. The two-tailed P<0.05 was used to define statistical significance. Statistical analysis was performed using SPSS version 17.0 software.

### Reproducibility

Intraobserver and interobserver variability were assessed on separate occasions, using new arbitrary images for SS, s-SR, e-SR and a-SR, blinded to the previous results. Ten subjects were randomly selected from each group for the analyses. For the interobserver variability assessment, the first observer performed the analyses. Second observer repeated the analyses within 24 hours. For assessment of the intra-observer variability the analyses were repeated twice by the first observer within 1 week. Results were analyzed using coefficient of variation where differences between measurements were expressed as the ratio of the standard deviation to the means and multiplied by 100.

## Results

### Characterization of study population

The clinical features of different groups were presented (Table 1). No one was lost to follow-up. There were no significant differences in age, height, systolic, and diastolic blood pressure between groups. Heart rate, weight, and BSA of pregnant women increased statistically during pregnancy, and recovered after pregnancy.

**Table 1:** Clinical features of the groups.

Variable	Control (n = 50)	Pregnant women (n = 50)	Postpartum women (n = 50)	*P* value
Age(yrs)	27.76±2.87	28.64±2.50	28.64±2.50	NS
HR(bpm)	76.30±5.27	79.26±4.31† ‡	75.12±5.24	<0.05
Height(cm)	162.96±4.60	163.04±4.31	163.04±4.31	NS
Weight(kg)	56.18±5.89	72.00±4.97† ‡	55.24±3.89	<0.05
BSA(m2)	1.69±0.10	1.89±0.09† ‡	1.68±0.07	<0.05
SBP(mmHg)	117.46±6.58	119.70±6.29	119.78±5.57	NS
DBP(mmHg)	72.80±5.70	73.16±4.48	72.38±4.07	NS

Values represent the mean ± SD

^†^Control vs. pregnant group, P<0.05

^‡^Pregnant vs. postpartum group, P<0.05

^‡‡^Control vs. postpartum group, P<0.05

HR = heart rate; BSA = body surface area; SBP = systolic blood pressure; DBP = diastolic blood pressure

### Parameters of LV function

LVDd, EDV, ESV, SV, CO, Septal A´, Lateral E´ and Lateral A´ increased during the third trimester. EF, A and E/Septal E´ did not change statistically. E/A, Septal E´ and E/Lateral E´ decreased during the third trimester. There was no significant difference between the control and postpartum groups (Table 2).

**Table 2:** LV function parameters.

Variable	Control (n = 50)	Pregnant women (n = 50)	Postpartum women (n = 50)	P value
LVDd(mm)	44.02±2.58	46.26±2.21† ‡	43.24±2.34	<0.05
EDV(ml)	87.66±10.95	97.54±10.48† ‡	84.14±10.63	<0.05
ESV(ml)	29.90±4.59	34.46±5.52† ‡	28.68±4.13	<0.05
SV(ml)	57.76±8.93	63.08±6.77† ‡	55.46±7.71	<0.05
CO(L/min)	4.42±0.81	5.00±0.59† ‡	4.16±0.59	<0.05
LVEF (%)	65.77±4.18	64.75±3.31	65.89±2.97	NS
E wave(m/s)	0.98±0.13	0.91±0.14† ‡	0.98±0.13	<0.05
A wave(m/s)	0.55±0.08	0.55±0.09	0.55±0.09	NS
E/A	1.79±0.20	1.65±0.23† ‡	1.80±0.25	<0.05
Septal E´(cm/s)	13.10±1.87	12.20±1.59† ‡	13.20±1.73	<0.05
Septal A´(cm/s)	8.46±1.85	9.46±1.52† ‡	8.22±1.58	<0.05
E/ Septal E´	7.54±1.12	7.51±1.15	7.50±1.20	NS
Lateral E´(cm/s)	17.52±1.93	18.72±2.35† ‡	17.62±1.98	<0.05
Lateral A´(cm/s)	8.26±1.64	9.08±1.89† ‡	8.08±1.61	<0.05
E/ Lateral E´	5.63±0.99	4.92±0.98† ‡	5.63±1.10	<0.05

Values represent the mean ± SD

^†^Control vs. pregnant group, P<0.05

^‡^Pregnant vs. postpartum group, P<0.05

^‡‡^Control vs. postpartum group, P<0.05

LVDd = left ventricle end-diastolic dimension; EDV = end-diastolic volume; ESV = end-systolic volume; SV = stroke volume; CO = cardiac output; LVEF = left ventricle ejection fraction

### Conventional echocardiography parameters of LA function

Comparing the control and pregnant groups, LAAD, LAVmax, LAVmax/BSA, LAVpre-a, LAVpre-a/BSA, and LAVmin were increased in pregnant patients, although LAVmin/BSA did not significantly change. LA reservoir function, which was assessed by LA filling volume, LA expansion index, and LAEF, increased during pregnancy. LA conduct function, which was assessed by passive atrial stroke volume, passive emptying index, and LA conduit volume, decreased during pregnancy. LA booster pump function, which was assessed by active atrial stroke volume and active emptying index, increased during pregnancy. Changed parameters recovered to their normal values postpartum (Table 3).

**Table 3:** Conventional echocardiography parameters of LA function.

Variable	Control (n = 50)	Pregnant women (n = 50)	Postpartum women (n = 50)	*P* value
LAAD(mm)	30.32±2.86	35.34±1.85† ‡	31.16±2.07	<0.05
LAVmax(ml)	31.90±4.74	45.14±3.79† ‡	32.52±2.76	<0.05
LAVmax/BSA(ml/m2)	18.92±3.05	23.88±2.28† ‡	19.35±1.50	<0.05
LAVmin(ml)	13.92±2.84	16.66±2.48† ‡	14.54±2.12	<0.05
LAVmin/BSA(ml/m2)	8.23±1.65	8.79±1.23	8.65±1.20	NS
LAVpre-a(ml)	20.58±4.02	35.08±3.12† ‡	21.40±2.66	<0.05
LAVpre-a/BSA(ml/m2)	12.20±2.49	18.54±1.65† ‡	12.73±1.54	<0.05
LA filling volume(ml)	17.98±3.77	28.48±3.44† ‡	17.98±2.47	<0.05
LA expansion index	135.68±41.68	175.50±39.03† ‡	127.11±29.94	<0.05
LAEF (%)	56.25±7.80	63.05±4.77† ‡	55.25±5.68	<0.05
Passive volume(ml)	11.32±2.45	10.06±2.26† ‡	11.12±2.18	<0.05
Passive emptying index	35.70±6.53	22.21±4.19† ‡	34.22±6.19	<0.05
LA conduit volume(ml)	39.78±8.47	34.60±6.00† ‡	37.48±7.40	<0.05
Active volume(ml)	6.66±3.01	18.42±2.92† ‡	6.86±1.73	<0.05
Active emptying index	31.71±12.01	52.43±6.27† ‡	31.87±6.89	<0.05

Values represent the mean ± SD

^†^Control vs. pregnant group, P<0.05

^‡^Pregnant group vs. postpartum group, P<0.05

^‡‡^Control vs. postpartum group, P<0.05

LAAD = left atrial anteroposterior dimension; LAVmax = left atrial maximum volume; LAVmin = left atrial minimum volume; LAVpre-a = left atrial preatrial contraction volume; LAEF = left atrial ejection fraction.

### Strain and strain rate of LA using 2DSTE

SS, s-SR and absolute value of a-SR increased during pregnancy, opposite to the absolute value of e-SR (Table 4). There was no significant difference between postpartum and control groups.

**Table 4:** Strain and strain rate of LA using 2DSTE.

Variable	Control (n = 50)	Pregnant women (n = 50)	Postpartum women (n = 50)	*P* value
SS (%)	33.09±6.60	37.55±2.93† ‡	32.94±6.48	<0.05
s-SR (s-1)	1.91±0.25	2.25±0.31† ‡	1.96±0.24	<0.05
e-SR (s-1)	-2.36±0.35	-2.17±0.58† ‡	-2.36±0.32	<0.05
a-SR (s-1)	-1.59±0.26	-1.75±0.22† ‡	-1.55±0.27	<0.05

Values represent the mean ± SD

^†^Control vs. pregnant group, P<0.05

^‡^Pregnant group vs. postpartum group, P<0.05

^‡‡^Control vs. postpartum group, P<0.05

SS = systolic strain; s-SR = systolic strain rate; e-SR = early diastolic strain rate; a-SR = late diastolic strain rate

### Correlation analyses

In pregnant women, SS positively correlated with LA filling volume (r = 0.593, P = 0.001), LA expansion index (r = 0.528, P = 0.001), and LAEF (r = 0.580, P = 0.001). s-SR positively correlated with LA filling volume (r = 0.849, P = 0.001), LA expansion index (r = 0.650, P = 0.001), and LAEF (r = 0.631, P = 0.001). Nevertheless, e-SR negatively correlated with passive atrial stroke volume (r = -0.779, P = 0.001), passive emptying index (r = -0.763, P = 0.040), and LA conduit volume (r = -0.681, P = 0.001). a-SR negatively correlated with A wave (r = -0.822, P = 0.001).

### Reproducibility

The confidence intervals and percent change in mean for interobserver and intraobserver variability for SS, s-SR, e-SR and a-SR are shown (Table 5).

**Table 5:** Confidence intervals and percent change in mean for interobserver and intraobserver variability for SS, s-SR, e-SR and a-SR.

	Interobserver		Intraobserver	
Parameter	Change in mean (%)	95% CI	Change in mean (%)	95% CI
SS (%)	5.20	±1.93	6.05	±1.88
s-SR(s-1)	6.95	±3.90	7.15	±3.00
e-SR(s-1)	-8.17	±3.15	-8.97	±3.75
a-SR(s-1)	-6.78	±3.21	-7.09	±2.66

SS = systolic strain; s-SR = systolic strain rate; e-SR = early diastolic strain rate; a-SR = late diastolic strain rate; CI: Confidence interval.

## Discussion

**Fig 2:**
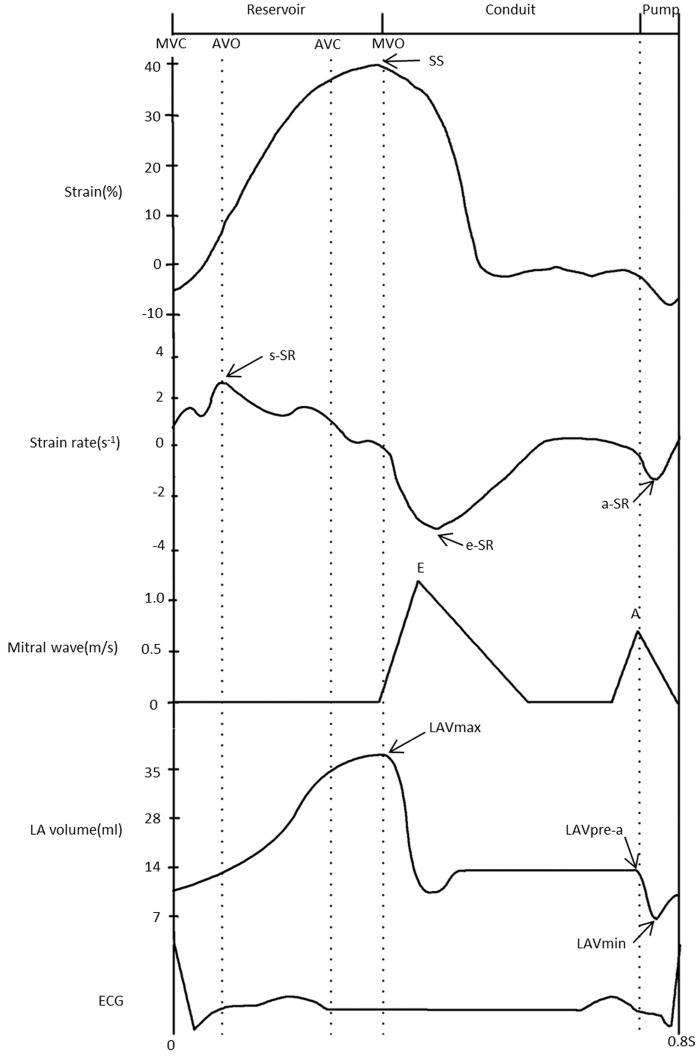
The relationship of time between LA strain, LA strain rate, mitral wave, LA volume, and ECG in the control group. LA = left atrial; SS = systolic strain; s-SR = systolic strain rate; e-SR = early diastolic strain rate; a-SR = late diastolic strain rate; MVC = mitral valve closure; AVO = aortic valve opening; AVC = aortic valve closure; MVO = mitral valve opening; LAVmax = LA maximum volume; LAVpre-a = LA preatrial contraction volume; LAVmin = LA minimum volume; ECG = electrocardiogram.

The major findings in this study were as follows: 1) the relationship of time was revealed in LA strain, LA strain rate, mitral wave, LA volume, and ECG parameters (Fig 2); 2) changes of LA function in each group were reliably assessed using LA strain and strain rate, which could be obtained by 2DSTE; 3) the second finding was confirmed using conventional echocardiography.

During pregnancy, enlargement of the LV satisfied physiological needs. The increase in CO was caused by increasing HR and SV, although EF did not change. We also found that E wave and E/A decreased, whereas the A wave remained unchanged. Similar results have been previously reported [12].

### LA reservoir function

LA reservoir function is defined based upon LA as a chamber to reserve blood return from the pulmonary vein and to store energy in the form of pressure during LV systole. This function of the left atrium is an important factor influencing cardiac output [13]. LA reservoir function is determined by LA myocardial contraction and relaxation and mitral annulus displacement during left ventricular contraction [6]. LA reservoir function can be represented by LA filling volume, LA expansion index, LAEF, SS, and s-SR [14]. In this study, we found that LA filling volume, LA expansion index, and LAEF positively correlated with SS and s-SR. Both groups of parameters were increased, which doubly confirmed that LA reservoir function was increased. The increase may be because: Both the decrease in LV compliance and increasing heart rate had a negative influence on LA emptying. LA began to adaptively enlarge to maintain adequate LV filling and satisfy increasing cardiac output [15].

### LA conduit function

LA conduit function is defined based upon LA as a conduit to allow the blood to drift into the LV from the pulmonary vessel during LV diastole. LA conduit function is mainly determined by the rate of left ventricular relaxation [16, 17]. LA conduit function can be represented by passive atrial stroke volume, passive emptying index, LA conduit volume, and absolute value of e-SR [14]. In this study, we found that passive atrial stroke volume, passive emptying index, and LA conduit volume positively correlated with the absolute value of e-SR. Both groups of parameters decreased, which doubly confirmed that LA conduit function was decreased. This decrease may be because: Physiological myocardial hypertrophy caused the decrease of LV relaxation, which could be reflected by decrease of E/A and E/Lateral E´ [1, 2].

### LA booster pump function

LA booster pump function is primarily as the active pump to maintain LV filling during LA systole. LA booster pump function is dependent not only on preload stretch (LAVpre-a) but also on afterload which is represented by LV end-diastolic pressure [18, 19]. LA booster pump function can be represented by active atrial stroke volume, active emptying index, and absolute value of a-SR [14]. First, we found that all of these parameters were increased. This could confirm that LA booster pump function is enhanced. This increase may be because, according to the Frank—Starling mechanism, enhancement of LA contractility is a response to increasing of LA preload [20], and is still in a compensatory state during normal pregnancy. Second, we also found that the absolute value of a-SR positively correlated with the A wave. A previous study found that the A wave increased only in the first two trimesters and returned to normal in the third trimester [12]. Compared with the A wave, the absolute value of a-SR may be a more sensitive parameter to assess LA booster pump function. Lastly, we found no connection between active atrial stroke volume, active emptying index, and absolute value of a-SR in this study. This requires further research.

After delivery, LA function in pregnancy returned to normal. This suggests that changes during pregnancy are reversible.

### 2DSTE

After assessing for reproducibility, we found that 2DSTE is a repeatable assessment of LA function. 2DSTE overcomes the shortcomings of tissue Doppler imaging. LA Strain and strain rate, which were obtained by 2DSTE, may be more sensitive than pulse wave Doppler of mitral valve during pregnancy.

### Limitations

Several limitations in this study should be addressed. First, the LA is farther from the transducer in the apical views, and the LA myocardium is thinner than the LV myocardium. These have a negative impact on the acquisition of high-quality 2D images and tracking the speckle. Second, we did not research LA function in pregnant women during the first and second trimesters due to the influence of the insurance policy. This will be the primary aim of future studies. Third, LA pressure, pulmonary arterial pressure, and their relationship with LA function were not assessed, which may explain the palpitations, chest pressure, and dyspnea. Last, only Asian population enrolled in this study which had a small sample size.

## Conclusions

We demonstrated that the reservoir and booster pump functions of the LA increased, and conduit function decreased during pregnancy. The changes seen during pregnancy were reversible. 2DSTE correlates well with the conventional method in assessment of LA function, and can been easily and repeatedly applied in the clinical setting.
